# Grapes

**Published:** 1862-05

**Authors:** 


**GRAPES.**—The grape is delicious to the taste and nutritious to the system; its health-promoting and disease-dispersing powers are such as to have attracted the attention of medical men in different countries. "On the banks of the Blue Mozelle" its remedial virtues are considered to be of sufficient value to warrant the establishment of a "grape-cure," by Dr. Hezpin, of Metz. The treatment lasts from three to six weeks, and the amount to be taken is from two to four pounds every day. We certainly would prefer a "course of grapes" of this kind to any "course of medicine" ever heard of. It would do many a child more good than a "course of sprouts." There can not be the slightest doubt that many a patient who has exhausted all the "pathies" yet known, in the vain hope of regaining health, would get perfectly well, if compelled to live on what grapes he could himself raise, using them as his only food and medicine for four months in the year. Let every one, then, who has the permanent control of one square yard of unoccupied ground, in city, in town, or country, plant a vine thereon, and dig about it, and dress it, and enrich it from time to time. It will be a delightful recreation for many an otherwise unoccupied hour, and will bring its full reward. Why so valuable a product has not met with more attention in this country, arises most probably from the fact that—as our English cousins with too much truth have said of us—"Americans won't engage in any thing which can not be completed in twenty minutes." But although it requires several years for a grapery to produce a full yield, that product is so rich that it ought to encourage the planting of a vineyard, even if but a dozen vines, in every farm of an acre or over. It is very certain that the best varieties of grapes can be produced for two cents a pound, on land costing one hundred dollars an acre. Uncounted tons could be sold in New-York City any year at six cents a pound, by the hundred.

A poor girl in California picked up the cutting of a grape-vine, thrown into the road, in order to drive her mule with. She carried it home, and though it was wilted and worn and appeared good for nothing, she stuck it into the ground. "It has a little life left," she said; "I will try and save it." So she watered it, and watched it, and trained it, and took as much care of it as if it were the most promising shoot in the world. In one year, after it was six years old, it bore five thousand bunches of grapes, and each bunch weighed one pound; these, on being sold, brought her the handsome sum of four thousand dollars.

There is scarcely any product of the earth which exceeds in variety and value that of the grape for the amount of labor bestowed upon it. As an esculent, it is perfectly delicious. Every table in the land should fairly groan under the liberal supply of an article of food so acceptable to the tastes of all people of all climes. Grapes require to ripen early in the autumn in the latitude of New-York City, and we gather them from our out-door vines until the first of December, up to which time they daily become sweeter. There seems to be a life within them when ripe which resists freezing when the weather is several degrees below the freezing-point. If properly packed and kept in a cool, dry place, they may be preserved in excellent condition until spring. Every family having a rear-yard of their own, of even twenty feet square, would find it profitable and healthful to cultivate half a dozen grape-vines, the fruit of which would make a healthful dessert for a family for several months of every year. For such a purpose it is not exceeded by any thing which can be placed upon our tables. The delicate acid of the grape, in common with all fruits and berries, is ascertained beyond doubt to be a most efficient agent in acting on the liver and stimulating that secretion of bile without which, there can not be any health for twenty-four hours at a time. The grape, in consequence of the copiousness of its juice, is more beneficial in these respects than other fruits. If eaten without swallowing the pulp or seed, the juice immediately under the skin, being decidedly astringent in its nature, has a controlling effect in all cases of looseness of the bowels.

To that incredible number of persons who suffer from constipation, the grape eaten and swallowed, pulp, seeds and all, as a dessert after breakfast and dinner, possesses an efficiency, without any injurious effect whatever, which makes it one of the most valuable boons of nature; while, if the system is in a healthy condition, the combined effect of swallowing the entire fruit is to nourish, strengthen, and revive.

The vine is long-lived and very productive; that which Racine planted in the Rue des Marais, Paris, in 1699, was covered with fruit in 1855, which was a hundred and fifty years later. The lamented Downing "saw one Isabella vine which produced in one season three thousand fine clusters of well-ripened fruit." The average retail price of good grapes, in the hight of the season in New-York, is fourteen cents a pound. One good vine, occupying a space of four by six feet, will yield fifty pounds, which, at even ten cents a pound, amounts to a liberal reward for the outlay of capital and labor bestowed.

The cultivation of the grape invites the attention of farmers in general, while to the citizen, who spends his summer at his country-seat, returning, as such do, about the first of November, we think of no culture which combines so many advantages of pleasure, comfort, interest and health, as the cultivation of a vineyard, so immediately productive to himself, and promising increased benefits to his children and grandchildren after him.

